# Reaching a Consensus on the Definition of Telerehabilitation: World Federation of Neurorehabilitation Telerehabilitation Special Interest Group

**DOI:** 10.63144/ijt.2025.6708

**Published:** 2025-06-12

**Authors:** Annie J. Hill, Kirsten Stangenberg-Gliss, Yeşim Kurtais Aytür, Pamela Enderby, Claudine Auger, Ali Gamal, Carl Froilan Leochico

**Affiliations:** 1 Centre for Research Excellence in Aphasia Recovery and Rehabilitation, School of Allied Health, Human Services and Sport, La Trobe University, Melbourne, Australia; 2 University of Applied Sciences and Arts Hildesheim, Germany; 3 Ankara University Faculty of Medicine, Department of Physical Medicine & Rehabilitation, Turkey; 4 Department of Medicine and Population Science, University of Sheffield, United Kingdom; 5 Faculty of Medicine, School of Rehabilitation, Université de Montréal, and Centre for Interdisciplinary Research in Rehabilitation of Greater Montreal, Québec, Canada; 6 Alzaiem Alazhari University, Khartoum North, Technical Medical College, Physiotherapy Department, Khartoum, Sudan; 7 Division of Physical Medicine and Rehabilitation, Department of Medicine, University of Toronto, Toronto, Ontario, Canada; 8 Department of Rehabilitation Medicine, Philippine General Hospital, University of the Philippines Manila, Philippines; 9 Department of Physical Medicine and Rehabilitation, St. Luke’s Medical Center, Philippines

**Keywords:** Consensus, Consensus e-Delphi, Telerehabilitation

## Abstract

The research into and the adoption of telerehabilitation has greatly expanded over the last two decades. With this increasing level of interest in telerehabilitation there is a need for a comprehensive definition. The Telerehabilitation Special Interest Group of the World Federation of Neurorehabilitation is comprised of a diverse group of researchers from over 30 countries and so is well placed to reach a consensus on a definition of telerehabilitation and disseminate this widely. An e-Delphi approach was employed within the special interest group to reach a consensus on the definition. The agreed comprehensive definition of telerehabilitation includes a formal definition, an abbreviated version and a lay version, each with distinct purposes. A description of the scope of telerehabilitation is included, as well as an overview of the various modes of telerehabilitation. It is anticipated that this definition of telerehabilitation may assist researchers, clinicians, advocates and policy makers in a range of purposes.

In view of the advancements in and widespread adoption of telerehabilitation over the past two decades, a process that has been markedly accelerated by the pandemic caused by the severe acute respiratory syndrome (SARS-CoV-2) virus, there is an urgent need for a clear, consensus-driven definition that reflects current telerehabilitation practice. Publication of articles on telerehabilitation have exponentially increased during this period. For example, the Web of Science database reports containing just 25 articles on ‘telerehabilitation’ in 2005 and 716 articles in 2024 (WoS, 29.4.2025). This increase in research, publication and clinical practice of telerehabilitation, calls for terminology standardization as a means to promote clarity in research, policy, and clinical practice.

The World Federation of Neurorehabilitation’s Special Interest Group (SIG) on Telerehabilitation is uniquely positioned to lead this effort. Comprising international experts from a range of disciplines, including physiotherapy, occupational therapy, speech-language pathology, rehabilitation medicine, and health technology, the SIG brings together a diverse and relevant voice from over 30 countries. The SIG’s multidisciplinary and global composition ensures that any definition developed will be inclusive, contextually adaptable, and grounded in clinical reality. Furthermore, as a recognised body within the professional neurorehabilitation community, the SIG holds the credibility and influence necessary to promote widespread adoption of a standardised definition. The establishment of a unified understanding of telerehabilitation is imperative for the promotion of consistent clinical practice and equitable service delivery, as well as for the facilitation of robust research and policy development in the domain of telerehabilitation across diverse healthcare systems.

An additional need for an agreed upon definition of telerehabilitation arose from the work of other Telerehabilitation SIG working groups which are engaged in reviews of telerehabilitation guidelines and outcome measures. The use of a definition within this work better enables search and synthesis of findings. Furthermore, an agreed definition of telerehabilitation could help define the scope of the Telerehabilitation SIG and our contributions while working with other SIGs of the WFNR. Therefore, the aim of the Telerehabilitation SIG for this piece of work was to come to a consensus on a working definition of telerehabilitation and to disseminate this widely.

## Methods

An e-Delphi approach was employed to achieve the goal of a definition of telerehabilitation. An e-Delphi approach is an online method often used in research to strengthen decision-making processes and reach a consensus on that decision (Msibi et al., 2018). The method included formulation of the definition, critical appraisal of the definition, refinement of the definition and a final consensus vote.

A draft definition of telerehabilitation, considering the telerehabilitation and rehabilitation definitions previously used in the literature (Brennan et al., 2009; World Health Organisation, 2019), was circulated to all telerehabilitation SIG members ahead of the September 2024 meeting. The definition was discussed at the meeting and a call made for SIG members to volunteer to the Definition Working Group. A total of seven researchers representing seven countries across five continents (Europe, North America, Asia, Australia, Africa), participated in the Definition Working Group.

The lead researcher of the working group refined the working definition and circulated this to all working group members for review, suggestions and edits. The telerehabilitation definition working group then edited and refined the definition through email. A revised definition was then circulated to all Telerehabilitation SIG members for review and comment and was further discussed at the November SIG meeting. After further refinement a final definition was circulated to all SIG members (*n* = 75) in December 2024 and again in early January 2025 along with a link through which to cast their vote on the definition. Within the vote SIG members were asked if they approved version 4 of the definition and could respond - yes, no or unsure. Members could provide further suggestions or comments in a text box. The results of the consensus voting were reported back to members at the 14th January 2025 meeting.

## Results

The WFNR Telerehabilitation SIG members came to a consensus on the definition of telerehabilitation and additional components that may be useful in the explanation and understanding of telerehabilitation at the meeting held on 14th January 2025. Fifteen members of the Telerehabilitation SIG cast their vote on the definition with 14 members (93.3%) endorsing the definition and one voting ‘unsure’. The comprehensive definition of telerehabilitation follows below:

### Definition of Telerehabilitation

Telerehabilitation is the application of information and communications technology (ICT) to deliver rehabilitation services (See Cochrane definition of rehabilitation below) over a distance by linking a healthcare provider to a beneficiary, caregiver, or any person(s) responsible for delivering care to the beneficiary, for the purposes of screening, assessment, intervention, consultation/coaching and/or supervision/monitoring.

#### Cochrane Definition of Rehabilitation

In a health care context, rehabilitation is defined as a “multimodal, person-centered, collaborative process” (Intervention-general), including interventions targeting a person’s “capacity (by addressing body structures, functions, and activities/participation) and/or contextual factors related to performance” (Intervention-specific) with the goal of “optimizing” the “functioning” (Outcome) of “persons with health conditions currently experiencing disability or likely to experience disability, or persons with disability” (Population) (Cochrane Rehabilitation, 2016, about us section).

### Scope of Telerehabilitation

Integral to telerehabilitation is the delivery of clinical rehabilitation services over a distance that are guided, monitored, or modified by a rehabilitation provider for each unique beneficiary or clinical purpose. That is, there is a connection or data flow between the provider and beneficiary/caregiver. Emerging technologies that are paired with telecommunications technology for delivery of rehabilitation over a distance are considered telerehabilitation. For example, virtual reality conducted via telerehabilitation where the provider and beneficiary are separated by distance or wearables that use Artificial Intelligence (AI) to monitor and track progress on a rehabilitation task and sends data or alerts to the provider’s email are a well-defined models of telerehabilitation.

“Telerehabilitation” may also be known by these terms: telepractice, teletherapy, virtual care, or remote rehabilitation. Although definitions of these terms may vary depending on their source, the use of telerehabilitation is preferred. Telehealth is considered a broader term that encompasses remote delivery of a wide range of health services by a range of healthcare providers (e.g., telemedicine, telepsychiatry, telepsychology, telenursing etc).

Telerehabilitation forms part of a larger concept known as e-Health or Digital Health which are terms given to electronic processes and communication technology which supports healthcare practice. E-Health/Digital Health includes but is not limited to electronic medical records and technology-delivered self-managed consumer education and training (e.g., therapy software apps used independently at home with no data transfer to a rehabilitation provider). While integral to the provision of healthcare, e-Health/Digital Health are not within the scope of telerehabilitation.

### Telerehabilitation Models of Service Delivery

Telerehabilitation may encompass individual sessions, group sessions, specialist clinical consultation, and clinical training/supervision. The technologies that are associated with telerehabilitation include the clinical use of videoconferencing (both hardware and software), teleconferencing, email, and store and forward of clinical data. Telerehabilitation service delivery may be provided between individual sites or multiple sites. Telerehabilitation encompasses synchronous (real-time delivery) or asynchronous (delayed delivery) formats. These formats may be used in combination (hybrid) for individual sessions (e.g., high quality video captured during the videoconferencing session and sent for review with the client during the session) and/or across an episode of care (e.g., high quality video taken during a videoconferencing session is sent to specialist for review after the session, with a management plan provided at a later time). These format combinations may be used for a variety of reasons, such as: (a) to optimise healthcare provider decision making in the presence of unreliable infrastructure/connectivity (Keck & Doarn, 2014); (b) in preparation for synchronous telerehabilitation sessions; (c) beneficiary education/monitoring; (d) specialist provider opinions.

### Abbreviated Definition of Telerehabilitation

Telerehabilitation is the application of information and communications technology (ICT) to deliver rehabilitation services (see Cochrane definition of rehabilitation) over a distance by linking rehabilitation provider to a beneficiary, caregiver, or any person(s) responsible for delivering care to the beneficiary, for the purposes of screening, assessment, intervention, consultation/coaching and/or supervision/monitoring. Telerehabilitation encompasses synchronous (real-time delivery) or asynchronous (delayed delivery) formats or a combination of these formats (hybrid).

### Lay Version of Definition of Telerehabilitation

Telerehabilitation is the use of information and communication technology (e.g., phone/mobile phone, videoconferencing such as Zoom, Teams) to deliver rehabilitation services (for example, but not limited to, medical, physical, occupational, psychological, cognitive or communication therapies) over a distance. Telerehabilitation involves a person providing the rehabilitation service in one location and a person receiving the service (person needing the rehabilitation service) or, a care partner or someone else delivering care to the person in a different location. Telerehabilitation may involve screening, assessment, intervention, consultation or monitoring of the care. Telerehabilitation may occur in real-time (e.g., phone or video call) or in a delayed manner (e.g., text message, email) or a combination of both.

### Telerehabilitation SIG Call to Action

Through ongoing research, policy advancement, and enhancements in technology, telerehabilitation holds the promise of revolutionizing the delivery of rehabilitation services, ensuring they are more accessible and effective for a wide range of communities.

## Discussion

The definition of telerehabilitation was developed and endorsed by the Telerehabilitation SIG of the WFNR in January 2025. Although only 20% of SIG members voted in the online poll, all members had the opportunity to review and comment on the definition on multiple occasions.

This definition of telerehabilitation offers a shared framework to guide both clinical practice and research. It promotes consistent terminology, supports the design of interventions, and enhances comparability across studies and services. It should be noted that both the full and abridged definitions serve distinct purposes. The full version is better suited for academic publications, policy documents, and grant applications. The abbreviated version is suitable for clinical guidelines, presentations and educational materials. The utilisation of both versions is intended to support advocacy, fundraising and policy development. A lay version of the definition is included to support public education, patient engagement, and community outreach, especially where patient co-design or involvement is essential.

The definition delineates the scope of telerehabilitation, encompassing assessment, treatment, education, and follow-up. This clarifies its relevance in clinical models, which may be important in grant applications and health policy discussions. The scope section also provides guidance on modes of delivery, including synchronous, asynchronous, and hybrid models, which are employed in practice, teaching, and service planning. The definition can inform professional training and be cited in research and funding proposals. The scope section can also be used to differentiate between emerging technologies and this classic definition of telerehabilitation. It’s critical to comprehend the scope and extent of telerehabilitation by being aware of examples that may be perceived as telerehabilitation procedures but do not fit in this category. For example, the case where the patient follows an exercise program with guidance of an exercise video uploaded to the web, and the healthcare personnel calls at certain intervals to question his/her compliance with the exercise and whether any adverse effects are observed can not be considered within the scope of telerehabilitation, as there is no data flow between the care provider and care receiver. Use of technology-delivered self-managed consumer education and training (e.g., therapy software apps used independently at home with no data transfer to a rehabilitation provider) is not telerehabilitation.

However, it is important to note that the text is not without its limitations. The field of telerehabilitation is evolving, and the definition should be reviewed and revised periodically (e.g., every 5 years). Furthermore, it is pertinent to note that while this definition of telerehabilitation may be applicable to other fields, it was conceptualised within the field of neurorehabilitation. As such other fields may need to adapt elements of the definition for their specific purposes. Future reviews of the definition may include input from other fields that also use telerehabilitation.

The WFNR Telerehabilitation Special Interest Group invites contributions from the global community. Researchers and clinicians are invited to share comments and suggestions or join the SIG by contacting the leadership team at telerehab.wfnr@gmail.com.
